# Three-dimensional computational fluid dynamics analysis of an electric submerged arc furnace

**DOI:** 10.1038/s41598-021-96085-1

**Published:** 2021-09-03

**Authors:** K. Karalis, N. Karalis, N. Karkalos, Ν. Ntallis, G. S. E. Antipas, A. Xenidis

**Affiliations:** 1 Institute of Geological Sciences, University of Bern, 3012 Bern, Switzerland; 2 Mechanical Engineer, Mining and Metallurgical Engineer, National Technical University of Athens, Athens, Greece; 3 School of Mechanical Engineering, National Technical University of Athens, Zografou Campus, 15780 Athens, Greece; 4 Molecular Modelling Laboratory, Park Innovaare, 5234 Villigen, Switzerland

**Keywords:** Computational methods, Fluid dynamics

## Abstract

A computational fluid dynamics (CFD) method is proposed to analyze the operation of a submerged electric arc furnace (SAF) used in ferronickel production. A three-dimensional mathematical model was used for the time-dependent solution of the fluid flow, heat transfer and electromagnetic phenomena. The slag's physical properties, which play a crucial role in the SAF operation, were previously determined using classical molecular dynamics simulations and empirical relationships. The analysis revealed that the main slag properties affecting SAF operation are density, viscosity and electrical conductivity—the latter two being mutually dependent. The high electrical conductivity values of the slag favor melting via the high Joule heat produced within the slag region. Calculation of the dimensionless Péclet and Reynolds numbers revealed that the slag velocities play a decisive role in heat transfer and further indicate that the slag flow is laminar. The average slag velocity calculated 0.0001 m/s with maxima in the vicinity of the electrodes.

## Introduction

The principal ferronickel production route involves reductive roasting of lateritic ores in rotary kilns towards formation of calcine^1^, which further undergoes excess-carbon smelting^2^ in megawatt electric arc furnaces (SAF)^3–5^. SAFs typically operate at temperatures as high as 2000 K^6^ under the effect of Joule heating maintained by several self-backing Söderberg electrodes^7^ which are continuously consumed via submersion into a slag melt^1,7^. FeNi recovery is achieved by continuous chemical reduction promoted by high-temperature (fast) metal/slag reaction kinetics, enhanced mass and heat transfer, and the slag bath's electromagnetic stirring buoyancy effects^7,8^ and low slag viscosity^9^. Intrinsically, however, metal recovery is dependent on the slag’s electrical conductivity (EC) and its effect on transport properties of the mesoscale^10–12^; slag EC itself is very sensitive to even fractional changes in the chemical concentration of the ore, which reflect on final nickel quality.

Ultimately, slag and ferronickel EC define the association between the chemical composition of the ore feed and the energy consumption of the SAF; this association constitutes the most substantial reductive smelting metric—exclusively determined by trial and error^1^—and an intrinsically multi-scale modeling problem which has not been addressed so far. To this extent, we previously reported the first step of a multi-scale approach, regarding the first principles’ prediction of mesoscale slag EC to within 10% of the experimentally determined value (81.1 S/m at 1773 K) for an industrial-grade reductive smelting implementation^6^. In the current study, we apply the pre-determined properties (based on the atomic order, atomistic modeling)^3,4,6,13^ on the development of a three-dimensional mathematical model to examine the effect of the main operational parameters (applied voltage, current density etc.) in the process efficiency. In the former models, the distribution of temperature, velocity, and density regarding the slag electrical and thermal conductivity was determined. Based on the obtained results, correlations based on the slag composition to the overall power consumption can be made.

## Methods

### CFD model formulation

Maxwell’s equations were solved throughout the three-dimensional CFD domain to account for heat generation due to the materials’ resistance to the flow of electric current (Joule heating). These equations consist of the two Gauss laws, the Faraday law and the Ampere law^14^. If the charge density is initially zero, it remains zero at all times thereafter. In the absence of free charge density and of an external magnetic field, these equations respectively revert to the following form^15^

1 $$\nabla \cdot \mathbf{E}=0$$

2 $$\nabla \cdot \mathbf{B}=0$$

3 $$\nabla \times \mathbf{E}=-\frac{\vartheta {\varvec{B}}}{\vartheta t}$$

4 $$\nabla \times \mathbf{B}={\mu }_{0}{\varvec{j}}+{\mu }_{0}{\varepsilon }_{0}\frac{\partial {\varvec{E}}}{\partial t}$$

where **E** (V/m) is the applied (external) electric field, μ_0_ (H/m) is the magnetic permeability, ${\varepsilon }_{0}$ is the permittivity of free space. In the case where ${\rm E}$ is constant, the term in the right side of Eq. (4), which is describing the displacement current, is vanishing. The magnetic field **B** [T] is describing by the equation

5 $$\mathbf{B}={{\varvec{B}}}_{0}+{\varvec{b}}$$

where **B**_**0**_ and **b** represent the contributions from the external and induced magnetic field, respectively; in the current model, **B**_**0**_ is by definition zero and we have previously shown **b** to be infinitesimal^7,8^ and may, thus, be ignored. We note that Eq. (4) is valid under the additional provision that charge mobility is sufficiently low.

Based on the electric field, the current density field, **j** (A/m^2^), was calculated from Ohm’s law for a fluid with a velocity field **u** (m/s), formally expressed as inside a magnetic field

6 $$\mathbf{j}=\sigma ({\varvec{E}}+{\varvec{u}}\times {\varvec{B}})$$

where σ (S/m) is the electrical conductivity and the term **u** × **B** is due to the induced field. The magnetic force (Lorentz force law) in a charge $Q$, moving with velocity ${\varvec{u}}$ in a magnetic field ${\varvec{B}}$ is^16^

7 $${{\varvec{F}}}_{{\varvec{m}}{\varvec{a}}{\varvec{g}}}={\varvec{Q}}\left({\varvec{E}}+{\varvec{u}}\times {\varvec{B}}\right)$$

The mathematical statement of electric local charge conservation law can be formulated in the equation of continuity in the form of^16^

8 $$\frac{\partial \rho }{\partial t}+\nabla \cdot {\varvec{j}}=0$$

where $\rho$ is the volume charge density and ${\varvec{j}}$ is the volume current density.

In Eq. (7), the applied electric field **E** is also expressed as the gradient of an electric potential, φ (V) by satisfying

9 $$\mathbf{E}=-\nabla \mathrm{\varphi }$$

and due to Eqs. (6)–(8) it arises

10 $${\nabla }^{2}\mathrm{\varphi }=\nabla \cdot (\mathbf{u}\times \mathbf{B})$$

The velocity field was computed via the momentum differential equations describing the convective motion of a fluid with variable density ρ(t) (the latter required in order to be able to resolve thermal buoyancy effects in the SAF) as^17^.

11 $$\frac{\vartheta }{\vartheta t}\rho +\nabla \cdot \left(\rho {\varvec{u}}\right)=0$$

and

12 $$\frac{\mathrm{\vartheta }}{\mathrm{\vartheta t}}\uprho \mathbf{u}+\nabla \cdot \left(\uprho \mathbf{u}\mathbf{u}\right)=-\nabla \mathrm{P}+\nabla \cdot \left(\mu \nabla {\varvec{u}}\right)+\mathrm{\rho g }+\mathbf{j}\times \mathbf{B}+{\mathrm{S}}_{\mathrm{u}}$$

where, P (Pa) is the pressure and μ (Pa·s) is the dynamic viscosity. In Eq. (12), the combined effect of the intensity of the magnetic field and of the current density yields the Lorentz force source ($\mathbf{j}\times \mathbf{B}$), the source term S_u_ modifies the momentum balance depending on completion of solid–liquid phase change and vice versa by dampening the velocity at the phase change interface (solid–liquid) so that it becomes that of the solidified phase after the transition^18^ and g is the gravity force. S_u_ is given by^18,19^

13 $${\mathrm{S}}_{\mathrm{u}}=\frac{{\left(1-a\right)}^{2}}{{a}^{3}+\varepsilon }{\rm A}_{mush}\left({\varvec{u}}-{{\varvec{u}}}_{{\varvec{s}}}\right)$$

where *α* represents the volume fraction of the liquid phase, A_mush_ and ε represent arbitrary constants respectively (A_mush_ should be large and ε small to produce proper damping)^18^ and **u**_**solid**_ is the velocity of the solidified material (m/s).

Finally, the SAF temperature field, **T** (K), obeys energy conservation^17^

14 $$\rho {C}_{p}\frac{\vartheta {\varvec{T}}}{\vartheta t}+\left(\rho {C}_{p}{\varvec{u}}\cdot \nabla \right)\mathbf{T}=\nabla \cdot \left(k\nabla \mathbf{T}\right)+\frac{\mathbf{j}\cdot \mathbf{j}}{\sigma }$$

where, ρ is the fluid density (kg/m^3^), C_p_ is the heat capacity (J/(kg K)), k is the thermal conductivity (W/(m K)) and σ is the electrical conductivity (S/m). The **j**·**j**/σ term in Eq. (14) represents the energy source (Joule heating)^20^, relating the flow of electric current, q

15 $$q=\frac{{\varvec{j}}\cdot {\varvec{j}}}{\sigma }$$

where σ is equal either to the slag’s ionic contribution computed via the Nernst-Einstein relationship based on our precursor MD structural modelling (electrical conductivity range between 27 to 233 S/m for a temperature range of 1473 to 1773 K) or set to FeNi literature value(s)^6,21^. The total heat induced by the three electrodes in the SAF is approximately 44 MW^7^, while the heat due to the radiation was determined to be approximately 1.87 MW (4% of the total heat produced). Since an amount of heat due to radiation re-enters the molten bath via reflection on the furnace dome, radiative contributions have been ignored.

### CFD simulations

Convergence was assumed when the discretized equations residual fell below a preset tolerance of 10^–6^. The coupled thermal/electromagnetic problem [i.e., the system of Eqs. (11)–(14)] was solved via the stationary direct solver Multifrontal Massively Parallel Sparse direct Solver (MUMPS)^22^. The grid of the SAF model consisted of 3,237,985 free tetrahedron mesh elements, with the worst element having a minimum quality of 0.7465; element quality for the tetrahedron mesh was defined as^22^,

16 $$\mathrm{q}1=\frac{72\sqrt{3}\mathrm{V}}{{\left({\mathrm{h}}_{1}^{2}+{\mathrm{h}}_{2}^{2}+{\mathrm{h}}_{3}^{2}+{\mathrm{h}}_{4}^{2}+{\mathrm{h}}_{5}^{2}+{\mathrm{h}}_{6}^{2}\right)}^{3/2}}$$

where V is the volume and h_1_-h_6_ are the side lengths of the tetrahedron. If q1 > 0.1 the mesh quality (skewness) is not expected to affect the quality of the solution^22^. All CFD simulations were performed with COMSOL Multiphysics^20^.

### Materials properties

The air, slag and ferronickel phases were considered as homogeneous fluid continua^7^. The density, viscosity and electrical conductivity of the slag layer and the electrical conductivity of the ferronickel layer were modeled as functions of temperature (see Table 1). For the slag, the values obtained by performing MD simulations while for the other domains the properties were obtained from the literature^10–12,23–28^.

**Table 1:** Thermophysical properties of materials used in the computations.

Properties	Slag	Ferronickel	Electrodes	Firebricks
Density [kg/m^3^]	7.48 × 10^−8^T^3^–3.799 × 10^−4^T^2^ + 0.25704 T + 3294.203	7000	1800	2300
Viscosity [kg/(m s)]	5 × 10^13^ T^−4.776^	0.005	–	–
Heat capacity [J/(kg K)]	1700	525	1800	1000
Thermal conductivity [W/(m K)]	1	15	18	1.22
Electrical conductivity [S/m]	5.34 (Τ < Τ_melt_) 427.32 – 0.866 T + 0.000515 T^2^ − 7.57E − 8T^3^ (T > T_melt_)	1E6−330.83 T	25,000	0.01
Solidus temperature [K]	1420	1570	−	−
Liquidus temperature [K]	1450	1600	−	−
Latent heat [J/kg]	400,000	290,000	−	−

The slag properties were determined via MD simulations^21^ while for the ferronickel, electrode and firebrick domains they were obtained from the literature^11,23–29^. A graphical representation of the density, viscosity and electrical conductivity is given in Appendix 1.

The slag properties were determined via MD simulations^6^ while for the ferronickel, electrode and firebrick domains they were obtained from the literature^11,12,23–28^. A graphical representation of the density, viscosity and electrical conductivity is given in Appendix.

## Results and discussion

By solving Eqs. (12)–(15), the values and the corresponding gradients of temperature and velocity were calculated in the developed three-dimensional discretized domain. The temperature gradients in the SAF are a direct outcome of Joule heating^6^ which, in turn, is proportional to the current density, j, relating the flow of electric current, q according to Eq. (14)^7,8^. To calculate j, the aggregate FeNi/slag σ value can be inputted into Eq. (5), then solving the system of Eqs. (5) and (10) in order to obtain the spatial distribution of the electric potential V, on the provisions that B is zero as there is no magnetic field external to the SAF rig, B_0_ is by definition zero [Eq. (6)] and b is negligible as previously shown by us^8^. In this manner we were able to examine whether the experimentally-observed electric SAF potential in the range 380–400 V may be reproduced by setting the applied electric current of the CFD model to values within the SAF operating parameter range of 68–72 kA.

**Figure 1:**
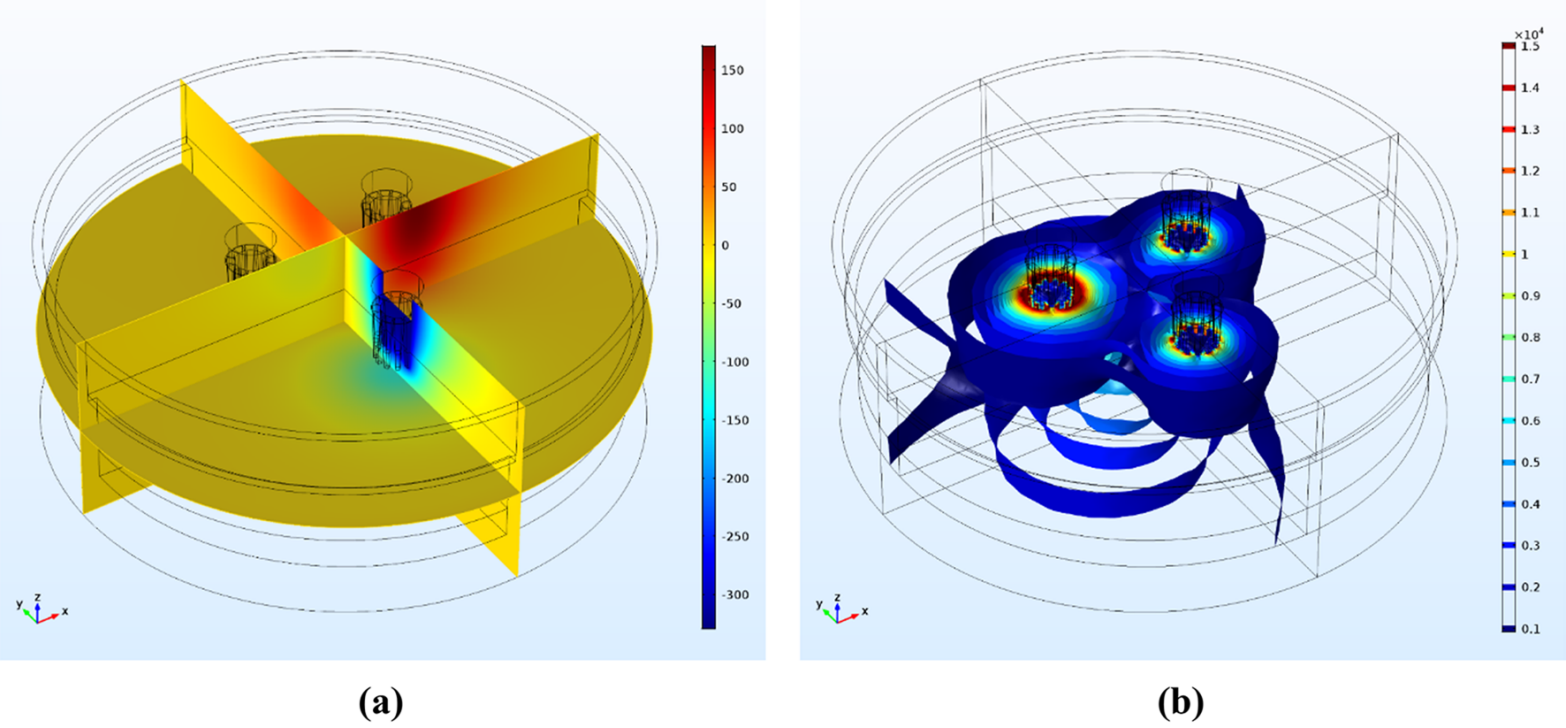
(**a**) Electric potential distribution (V) in the SAF extracted from a frame at 0.02 s. The minimum and maximum electric potential values are detected within the electrode core and a strong decay to ground values (0 V) is observed and (**b**) Current density isosurfaces (A/m^2^) at the time frame of 0.015 s. In comparison to the electric potential values, the current density is observed in both slag and ferronickel regions.

Hence, alternating current with a frequency of 5 Hz (see Appendix 1) and a maximum potential of 380 V was applied to the upper surface of the electrodes (V = 380 × sin(ωt + φ), φ equal to 0, 120 and 240 respectively for each electrode, Fig. 1). On the furnace freeboard, immediately above the air region (Fig. 1), a zero normal gradient for the electric potential was imposed^29–32^, in order to maintain the current densities and the joule heating phenomenon. In the firebrick-lined side walls adjacent to the air and slag layers, a constant heat-transfer coefficient of 100 W/(m^2^ K) and a free-stream temperature of 293.15 K were set^29^, equaling that of the water-cooled firebricks. In the firebrick side walls adjacent to the ferronickel layer, the temperature was set equal to that if the free stream and a constant heat-transfer rate of 10 W/(m^3^ K) were used, in accordance with the physically sensible value proposed in the literature^29,30^. At the bottom of the furnace, the temperature was considered constant and equal to 313.15 K (based on measurements performed at the LARCO plant) and the heat transfer-coefficient was set to 2 W/(m^3^ K). The initial SAF temperature was equal to 1000 K corresponding to the average temperature of the calcine (feed material). Using this temperature, the Joule phenomenon and the initial stages of melting were examined. Since the flow was treated exclusively within the region containing the slag, no-slip conditions (u = v = 0) needed to be imposed at the interfacial boundaries between both the slag and electrodes and the ferronickel and firebricks, while a slip boundary condition (v = 0) was imposed on the slag and air interfaces^30–33^. We note here that slip conditions ought to be applied in cases where viscous effects are negligible and there is no boundary layer, such as in a fluid/solid interface. As we determined previously^8^ the no-slip condition is particularly suitable for the interaction with a solid wall, when liquid layers adhere to a nearby solid boundary, due to infinite shear stress which reduces which velocity to zero, whereas slip boundary conditions are related to a stress-free condition in the gas–liquid interface.

### Distribution of electric potential

In order to understand the electric and thermal phenomena inside the SAF, we calculated the distribution of electric potential as portrayed in Fig. 1a on the time frame of 0.02 s across three cross sections. In the current frame the three electrodes are under potential values of -330 V, 330 V and 0 V respectively. Due to the ground potential applied in the sidewalls, the potential distribution inside the SAF lies between a minimum value of 0 V and a minimum/maximum of − 380 V and 380 V respectively. It is evident that the vast majority of the SAF’s sub-regions are under virtually zero electric potential, with the mere exception of regions in the immediate vicinity (up to 2.5 m) of electrode edges, which are affected by the applied potential in the three electrodes. This observation is on a par with very similar behavior observed based on our own precursor 2D analysis of the same SAF layout, having applied direct current (DC)^7,8^. Consequently, a qualitative agreement of electric potential distribution behavior is observed between 2 and 3D simulations.

**Figure 2:**
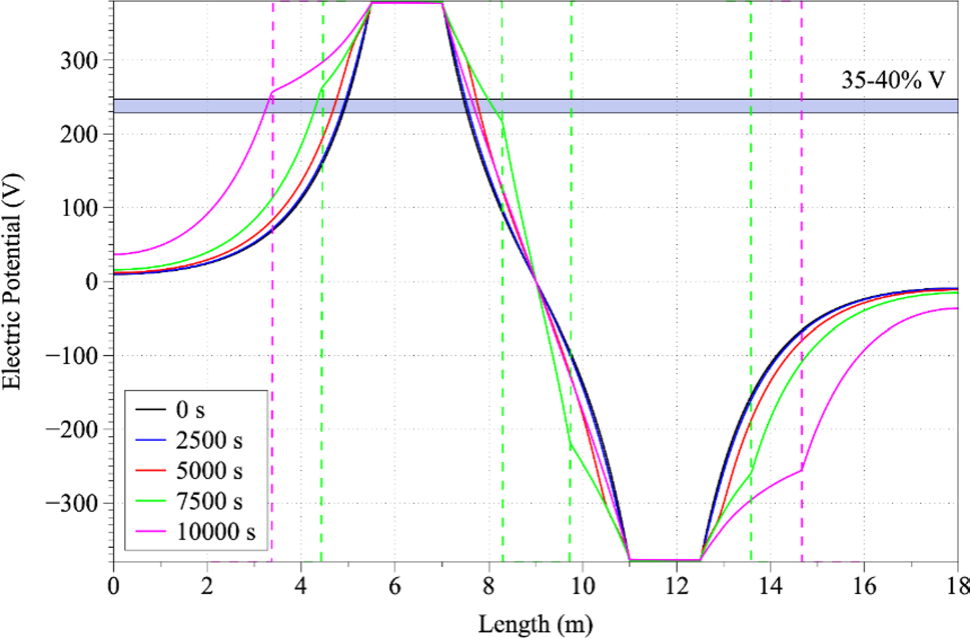
Electric potential distribution across an intercept line from x_1_ = 0 to x_2_ = 17 m for constant y = 1.7 m.

Based on the previous findings, and in order to understand the effect of the melting on the distribution of electric potential, we decided to investigate the evolution for the computational time periods of 0, 2500, 7500 and 10,000 s across an intercept line from x_1_ = 0 to x_2_ = 17 m for constant y = 1.7 m. The line chosen was 20 cm lower than the electrode tips in order to avoid spurious effects in the electrode corners (and to achieve smoother distribution, see Fig. 2). Along with the intercept, we were able to observe physically meaningful electric potential variations with respect to the selected time steps owing to the 5 Hz alternating current and to the temperature dependence of the electrical conductivity of the slag. In the later time steps (e.g. 5000 s) the electrical potential drop is smoother, which is due to the higher electrical conductivity values of the slag (cross-related to slag temperature). Aiming to determine the electric potential drop in respect to temperature, we focused our study on timesteps of 0 s and 2500 s, for which we observed a potential drop equal to 35–40% at distances of 50 cm from the electrode edge, while for 10,000 s the same pressure drop was determined to lie approximately 2.3 m from the electrode edge. Confirming model integrity, for timesteps of 5000 s and 7000 s, respectively, an electric potential drop of 20% in distances 20 cm from the electrode edges were calculated in accordance with the literature^29,30,34^. These observations are important because they offer a plausible explanation of the evolution of the Joule heat phenomenon and provide a firm theoretical basis for the explanation of furnace heating.

### Distribution of electric current density

**Figure 3:**
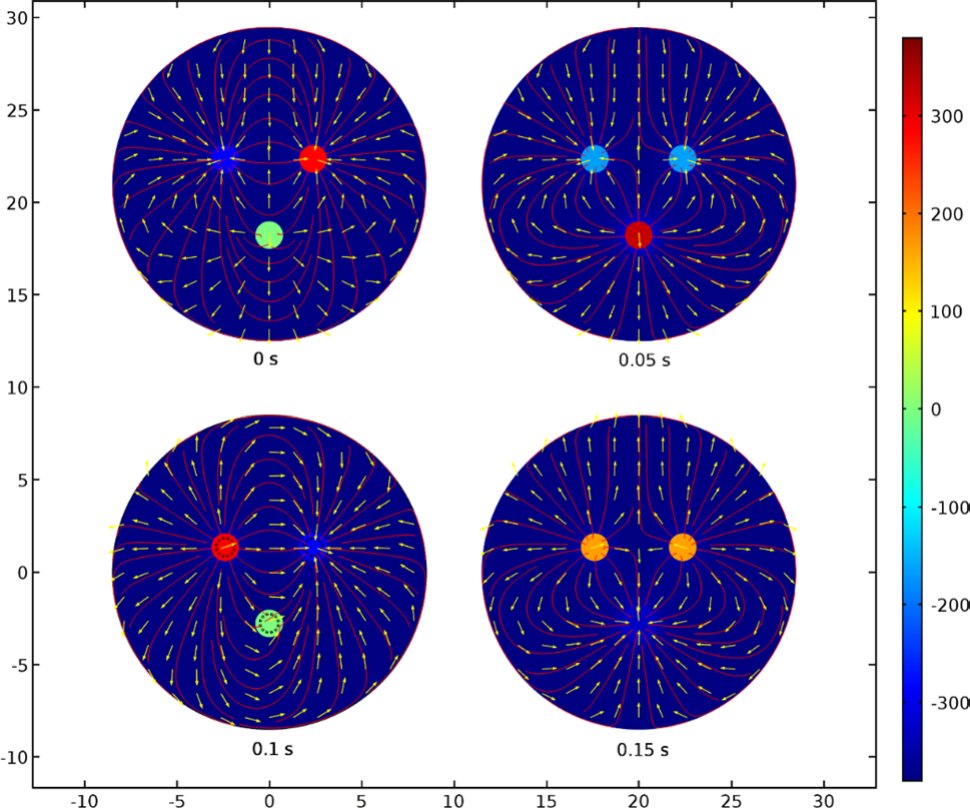
Current density pathways (A/m^2^) in respect to electrode potential.

In Fig. 1b a set of current density isosurfaces in the range 0.1–1.5 × 10^4^ A/m^2^ at a simulation time of 0.015 s is presented. In this snapshot, the left electrode was observed to be under a potential of 380 V while the other two electrodes had a potential of 190 V. The highest current densities observed were located in the slag phase, in the vicinity of the three electrodes. However, a fractional current density was also computed to be present in the ferronickel phase, suggesting that a small amount of heat is also produced. Figure 3 depicts the current density pathways in the upper layer of the slag phase (slag–air interaction layer) for several different simulation times. Due to the alternating current, where electrodes change their potential, it is evident that the direction of the electric current is following the conventional path, from the electrodes with a positive electric potential to those with a negative potential.

## Distribution of electric power: the Joule phenomenon

**Figure 4:**
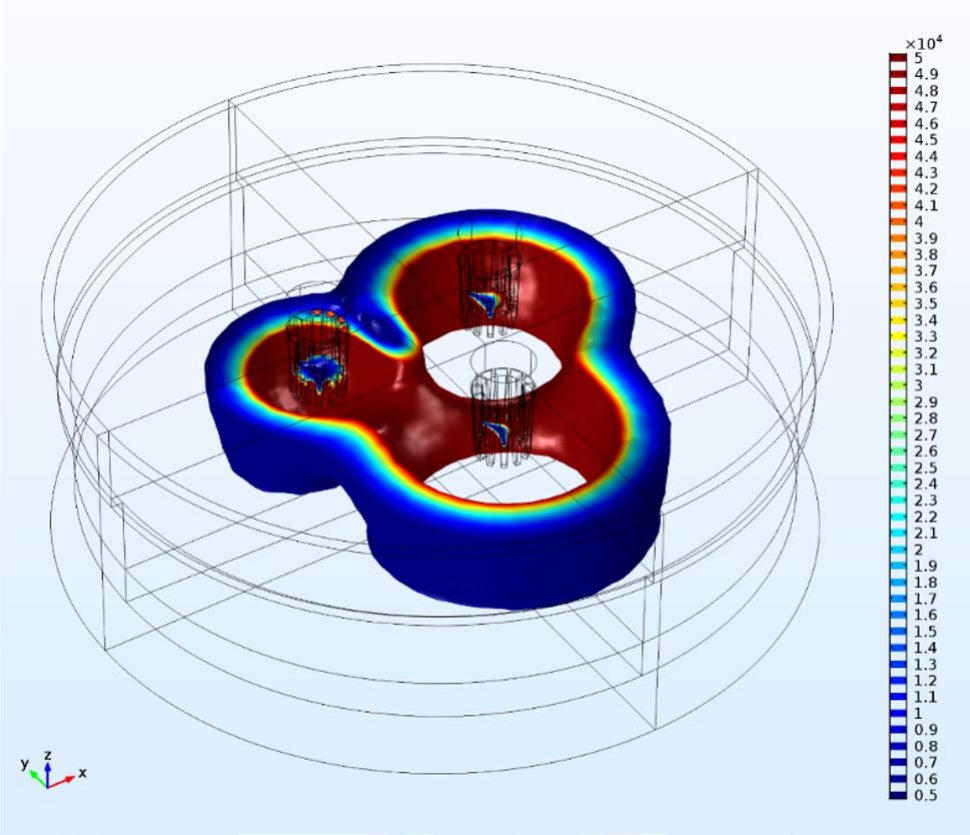
The reductive smelting in SAF is due to the Joule heat. The Joule heat isosurfaces (W/m^3^) at the time step of 0.007 s are presented. Due to the slag resistance in the electric current (small electrical conductivity) the maximum amount of heat is produced in the slag region.

Joule heating in the S facilitates feed material (calcine) melting and is proportional to the current density [Eq. (16)]. The higher amount of heat is produced in the electrodes' vicinity, resulting in temperature gradient in the bath; the buoyancy phenomenon is increased, leading to more efficient mixing. In Fig. 4, iso-surfaces of the Joule heat at 0.007 s are portrayed. The higher amount of heat is produced in the slag region close to the electrodes in full accordance with industrial observations where it also observed intense mixing due to higher velocities.

**Figure 5:**
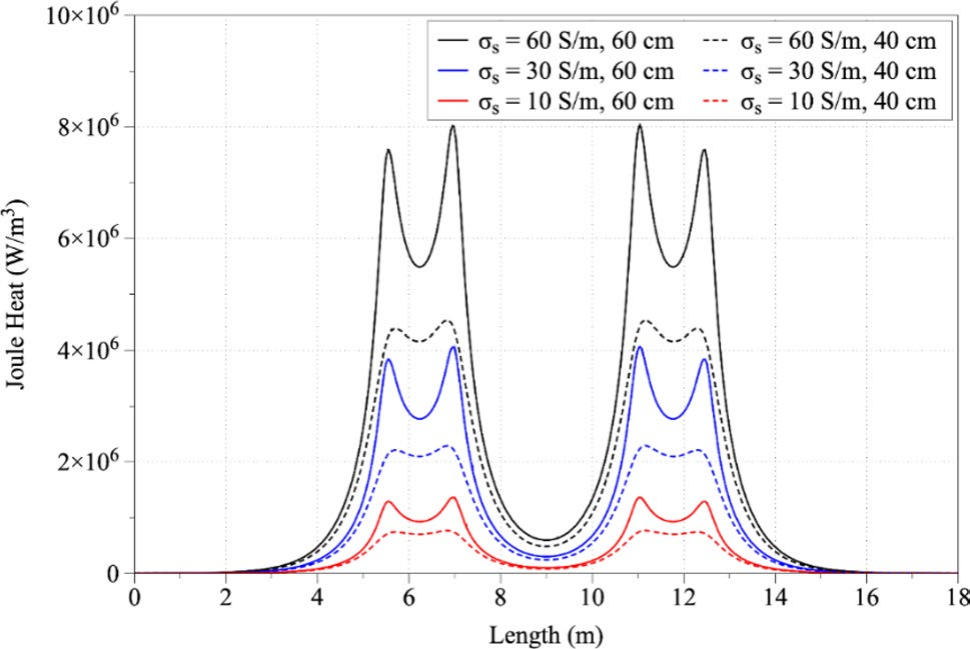
Joule heat distribution as a function of electrode immersion depth and electrical conductivity of the slag.

Figure 5 presents the Joule heat in respect to electrode immersion depths of 40 and 60 cm and to slag electrical conductivity values of 10, 30 and 60 S/m. To analyze the results, a cut line in the 3D geometry was drawn with coordinates x_1_ = 0 m until x_2_ = 18 m and constant y = 1.2 m. From the results, it is obvious that the higher values of the slag electrical conductivity lead to higher values of produced Joule heat. The increase of the immersion depth leads to higher amounts of Joule heat in the lower part of the slag region.

**Figure 6:**
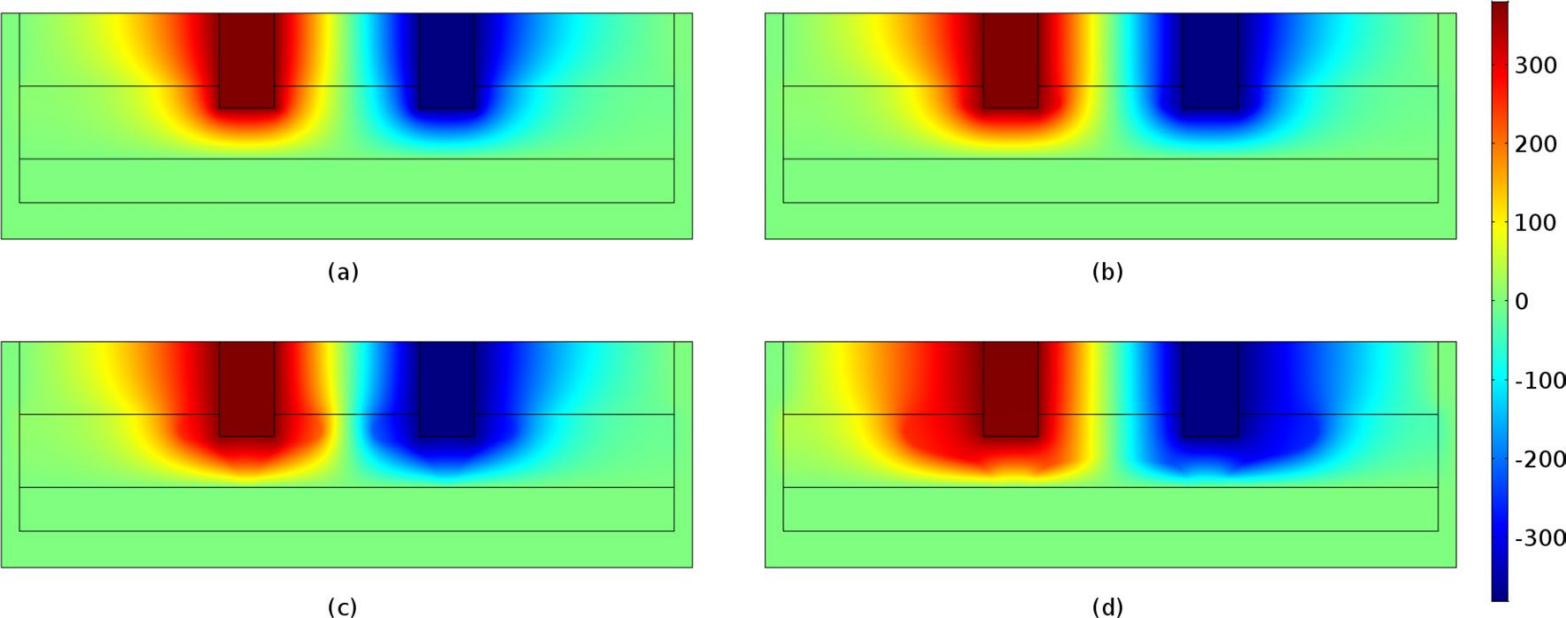
Potential distribution (V) at times equal to (**a**) 2500 s, (**b**) 5000 s, (**c**) 7500 s and (**d**) 10,000 s. The maximum absolute values are determined in the vicinity of the electrode tips and monotonically decreases to ground potential (0 V) values.

**Figure 7:**
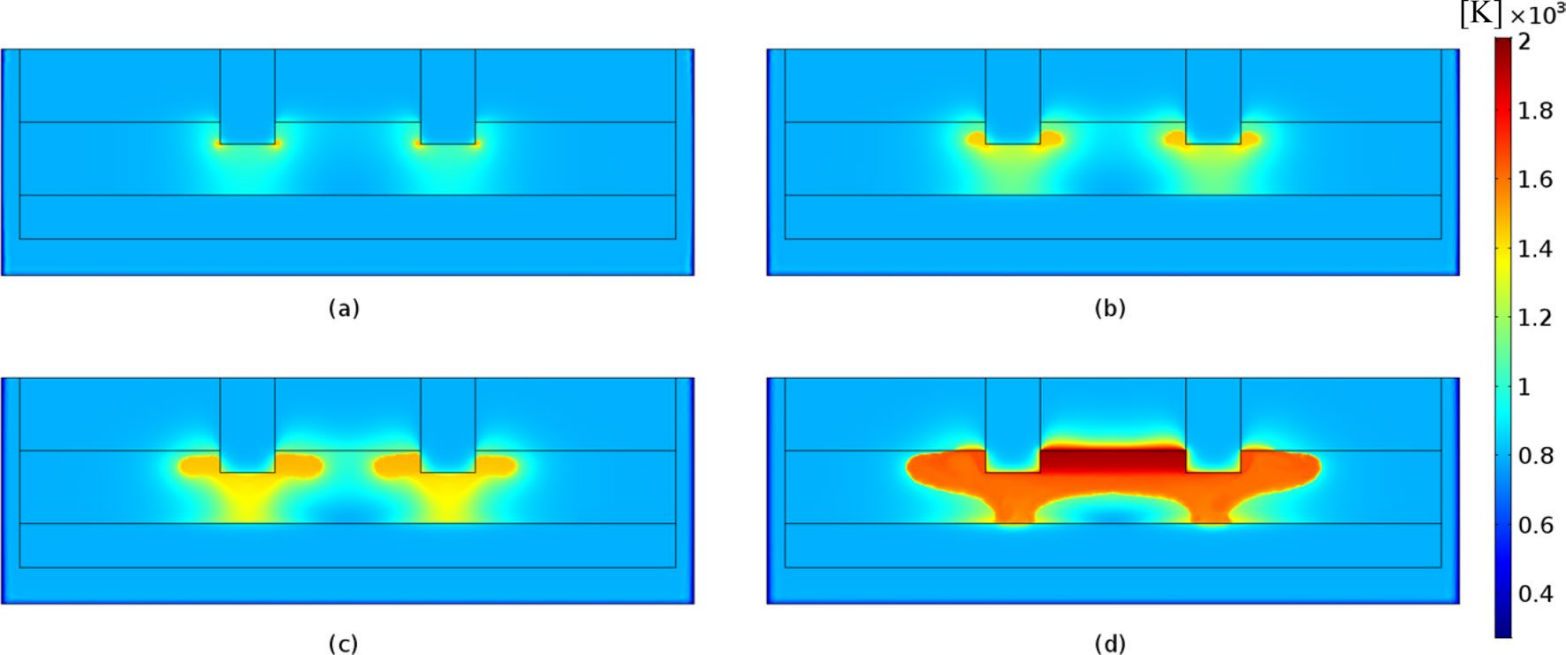
Temperature distribution (K × 10^3^) in the SAF at times equal to (**a**) 2500 s, (**b**) 5000 s, (**c**) 7500 s and (**d**) 10,000 s. A direct pathway from the immersed electrode tip to the ferronickel region is observed which can be defined as the submerged arc occurring in the SAFs.

**Figure 8:**
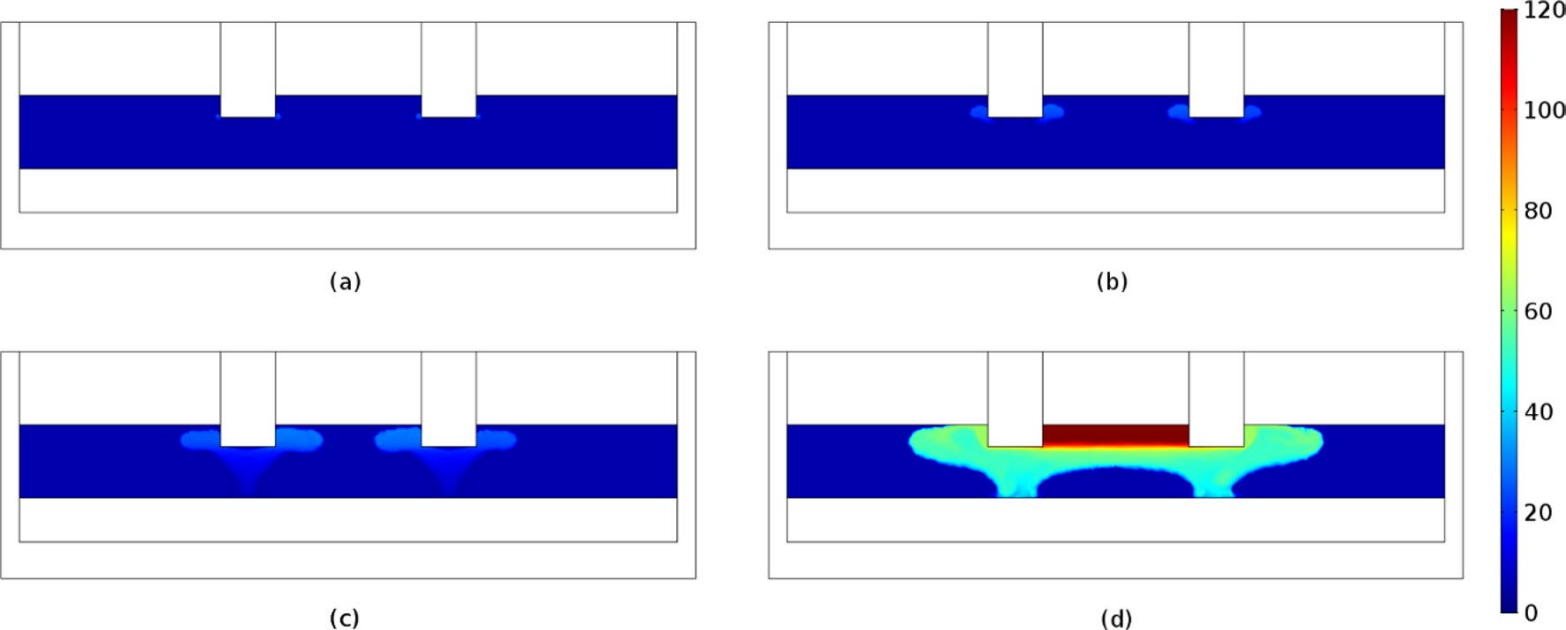
Electrical conductivity (S/m) of the slag phase at times equal to (**a**) 2500 s, (**b**) 5000 s, (**c**) 7500 s and (**d**) 10,000 s.

Figures 6 and 7 display the electric potential and temperature distribution for the time steps of 2500, 5000, 7500 and 10,000 s, respectively. Due to the temperature dependency of the slag's electrical conductivity, the electric potential distribution is modified with respect to the time. More specifically, the produced heat increases the temperature, which increases the electrical conductivity value, which according to Eqs. (14) and (15) leads to the production of higher amount of heat in the next timestep. As shown, the temperature distribution is correlated with the slag electrical conductivity values (Fig. 8). Increasing temperatures lead to correspondingly increasing electrical conductivities and vice versa. As may be observed, at a time step of 10000 s the temperature in the vicinity of the immersed electrodes is close to 2000 K, in accordance with industrial measurements performed at LARCO S.A. We performed measurements of the slag temperature via an infrared pyrometer which indicated that temperature varies between 1523 and 1633 K at the outlet of the SAF. Also, near the electrodes the temperature varies between 2273 and 3073 K^7^. Hence, initial slag melting occurs in the region between the electrodes, spreading radially thereafter. This phenomenon is due to the current density pathways as shown in Fig. 1b.

Based on the analysis with constant electrical conductivity values, it was determined that the higher slag electrical conductivity leads to higher Joule heat produced. The average Joule heat produced in the slag region is calculated equal to 1905.7 W/m^3^ and in the ferronickel equal to 0.27 W/m^3^. Increasing electrode immersion depth was determined to lead to higher Joule heat in both the slag and ferronickel phases. This is attributed to the fact that the electric current is transferred through the ferronickel region producing more heat in this region and hence responsible for temperature increase within this phase^35–38^. Through empirical relationships^10^ it was calculated that the thermal conductivity of the slag was in the range of 0.1 –1 W/(mK). Interestingly, any fluctuation of thermal conductivities of this order of magnitude did not appear to affect the outcome of the simulations.

### Distribution of slag density and velocity

**Figure 9:**
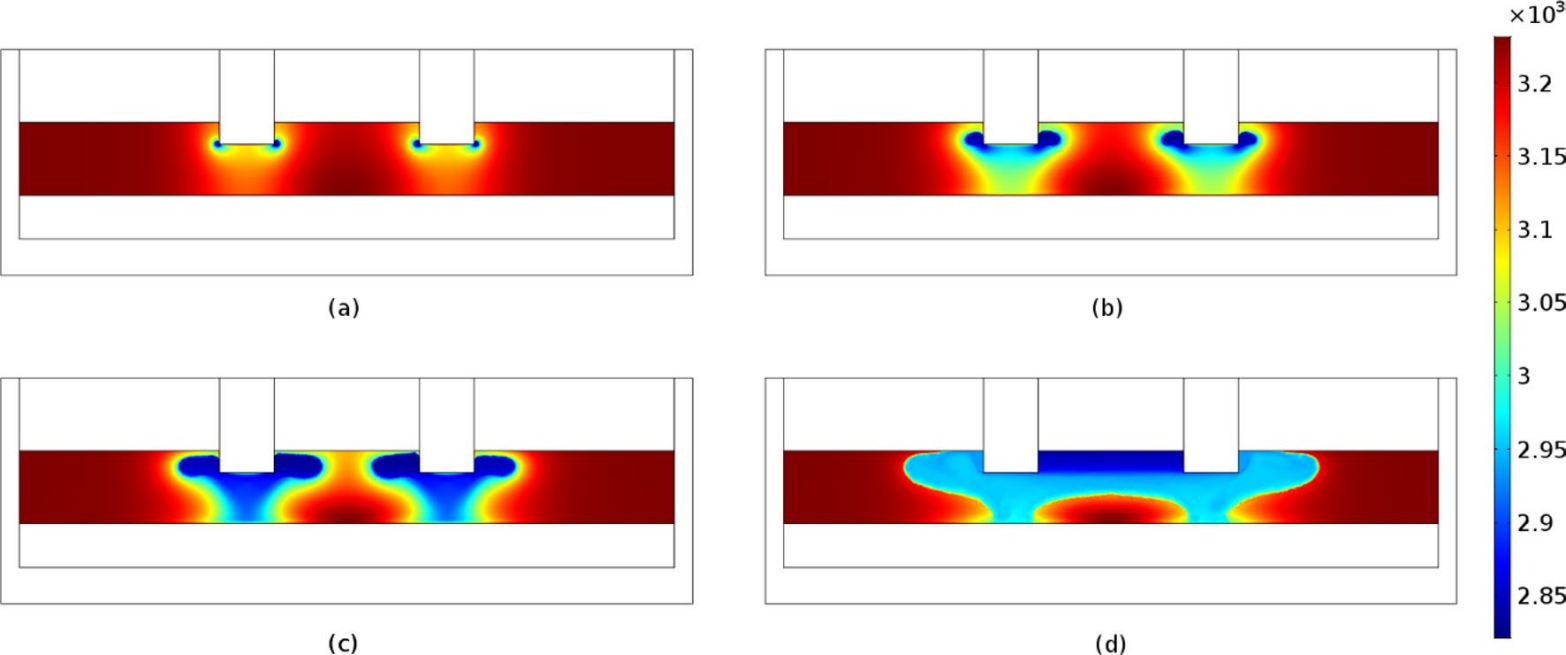
Distribution of slag density (kg/m^3^) with respect to times of (**a**) 2500 s, (**b**) 5000 s, (**c**) 7500 s and (**d**) 10,000 s. As expected, these snapshots are inversely proportional to the temperature distributions (Fig. 7).

Due to the variation of temperature in the slag phase, the density varies between 2800 kg/m^3^ in the vicinity of the electrodes to 3300 kg/m^3^ close to the firebricks (see Fig. 9). This variation was observed to lead to stirring of the slag bath due to buoyancy. We could hence deduce that slag melting is favored both from an increase in electrode potential as well as from increasing slag electrical conductivity. The latter is of crucial importance for the optimization of the SAF operation because additives can be used in the feed materials to artificially cause an increase of the electrical conductivity of the slag in a controlled fashion. For example, such additives are CaO and MgO which we have previously found that it acts by disrupting the alumino-silicate chains^4^. An optimum concentration of these additives can be determined by performing MD parametric analysis using the Buckingham-type transferable interatomic potential^6^.

**Figure 10:**
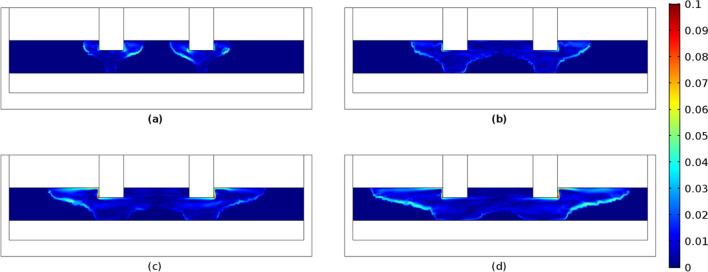
Velocity distribution (m/s) in the slag region with respect to times (**a**) 2000s, (**b**) 3000 s, (**c**) 4000 s and (**d**) 5000 s.

The maximum velocities in the vicinity of the electrodes and along the slag solid/liquid interface was found to be in the range 0.025–0.53 m/s^29,30,33,34,39,40^ with average values in the range 0.0001–0.028 m/s^29,39,41,42^. In Fig. 10, the velocity distribution is presented at times of 2000, 3000, 4000 and 5000 s, respectively. The maximum velocities—equal to 0.1 m/s—were detected on the solid/liquid interface of the slag as well as in the regions close to electrode surfaces. Increasing potentials and increasing slag electrical conductivity favor high velocities. For example, an indicative supporting case for this is determined by performing simulations with constant electrical conductivity; an increase in slag electrical conductivity lead to increased velocities. We also observed that this trend is independent of the use of AC current and of use of temperature-dependent electrical conductivities. Three dimensional simulations resulted in markedly lower velocities compared to two dimensional. This result may be attributed to the average Joule heat which is lower in comparison to 2D analysis. Consequently, 3D simulations yield smoother temperature profiles which lead to smaller density deviations and much milder buoyancy effects. However, as can be seen from the dimensionless Péclet number (computed to be excessively larger than unity, 231.3), the slag velocity plays an instrumental role in heat transfer-associated phenomena, in a flow which based on the associated Reynolds numbers (0.44) is characteristically laminar.

## Conclusions

In the current study a three-dimensional mathematical model describing the transient operation of an electric submerged arc furnace used in the ferronickel production was developed. The use of temperature dependent physical properties leads to instabilities which were results via the use of very small timesteps and fine mesh. One of the key physical properties affecting the operational efficiency which could lead to the reduction of the operational costs is the slag electrical conductivity; higher values lead to the production of higher amounts of Joule heat and consequently favors the smelting procedure. From the nondimensional analysis, it was revealed that slag flow is unambiguously laminar but with a decisive role in the heat transfer. The maximum velocities revealed in the vicinity of the three submerged electrodes.

## Supplementary Information

Supplementary Information.
