# Supplementary material for: Three-dimensional computational fluid dynamics analysis of an electric submerged arc furnace
Source: Sci Rep. 2021 Sep 3;11:17637. doi: 10.1038/s41598-021-96085-1 (PMC8417227; doi:10.1038/s41598-021-96085-1)
Supplement: Supplementary file 1 — Supplementary Information. [file 41598_2021_96085_MOESM1_ESM.docx]

**Three-dimensional computational fluid dynamics analysis of an electric submerged arc furnace**

**K. Karalis^1*^, N. Karalis^2^, N. Karkalos^3^, Ν. Ntallis^4^, G.S.E. Antipas^4^ and A. Xenidis^2^**

^1^Institute of Geological Sciences, University of Bern, CH-3012 Bern, Switzerland

^2^Mechanical Engineer, Mining and Metallurgical Engineer, National Technical University of Athens, MSc, PhD, Athens, Greece

^3^School of Mechanical Engineering, National Technical University of Athens, Zografou Campus, Athens 15780, Greece

^4^Molecular Modelling Laboratory, Park Innovaare, CH-5234 Villigen, Switzerland

*Email: konstantinos.karalis@geo.unibe.ch, Tel. +41 (0) 31 631 4564

# SUPPLEMENTARY INFORMATION


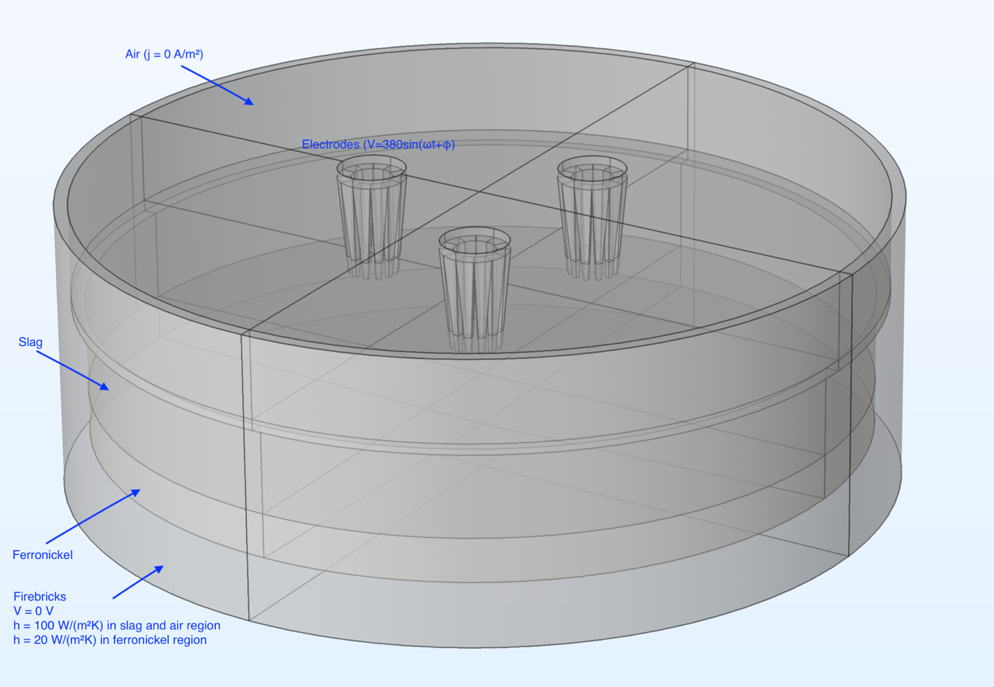


**Figure S1.** Geometry and boundary conditions of the EAF.

| 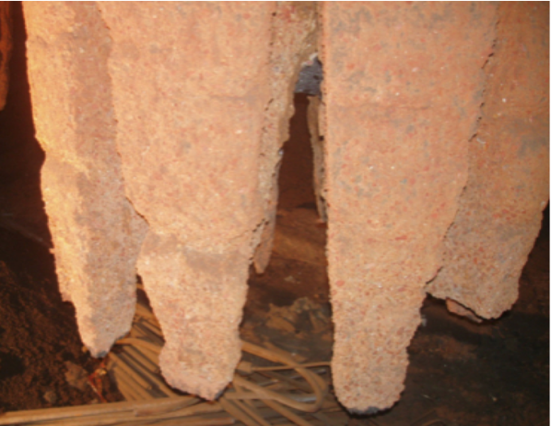 | 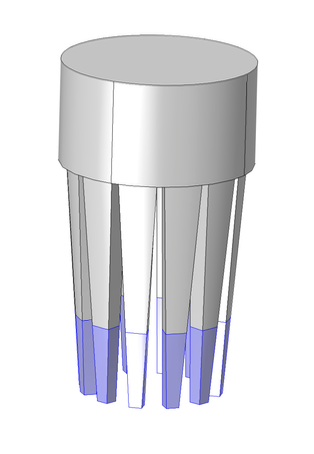 |
| --- | --- |
| **(a)** | **(b)** |

**Figure S2.** (a) Photograph of the Söderberg electrode during maintenance and (b) representation of the consumed electrode tip used in the simulations.

**Figure S3.** Temperature dependency of physical properties of slag.

**Appendix 1**

In the current mathematical model, a frequency of 5 Hz instead of 50 Hz (actually grid frequency) was used because the Joule heating in the low-frequency range (inclusive of the 5 to 50 Hz range) is frequency independent and acquiring a simulation time of 10000 seconds at 50 Hz is computationally intractable.

The slag electrical conductivity is known to not be frequency-dependent[^1^](#_ENREF_1), indicating that frequency will also not affect Joule heating. It has even been numerically proven that both the melt pool shape of the solidifying ingot and the distribution patterns of Joule heating and Lorentz force in the slag region are virtually unaffected by the applied AC frequency[^2^](#_ENREF_2). Also, from the slag’s skin effect and its capacitive reactance, it can be seen that the Joule heating is practically independent of AC frequency. The skin effect is expressed by the skin depth, δ, defined as[3](#_ENREF_3)

$\delta=\sqrt{2\rho/\omega\mu_{0}\mu_{r}}\sqrt{\sqrt{1+\left( \rho\omega\varepsilon_{0}\varepsilon_{r} \right)^{2}}+\rho\omega\varepsilon_{0}\varepsilon_{r}}$

where ρ is the specific resistivity (Ω*m), equal to 1/σ with σ the electric conductivity (S/m) ω is the angular frequency, μ_0_ is the magnetic permeability of vacuum, μ_r_ is the relative permeability of the material, ε_0_ is the electric permittivity of vacuum and ε_r_ is the relative permittivity of the material.

The factors of skin effect and capacitive reactance do not apply to melts (i.e. for slag temperatures above 1400K, the melting point)[^4^](#_ENREF_4). In case the system is not melted, the skin depth, δ, is defined by the distance at which the current density $j \sim j_{0}e^{-x/\delta}$ will reach a terminal value 1/e (~0.36)[^3^](#_ENREF_3), where x is the distance from the electrode tips. Effectively, δ expresses the reduction of the effective surface area through which electric current flows in any circuit. By substituting the values associated with the SAF geometry yields that the skin depths (meters) with respect to the slag electrical conductivity and AC frequency is 25.2-79.6 m (see Table S1).

| **σ (S/m)** | **5 (Hz)** | **50 (Hz)** |
| --- | --- | --- |
| 8 (T<1400K) | 79.6 | 25.2 |

**Table S1.** Skin depth (m) in respect to the slag electrical conductivity (S/m) and electrodes frequency (Hz).

According to this theoretical reasoning, at the melting point (T ~ 1400K, also see table above), the skin depth for AC frequencies of 5 and 50 Hz is 79.6 and 25.2 m, respectively. For these conditions, at distances of 0.5 and 1 m from the electrode tips (AC source), the deviation in the current density ($\boldsymbol{j}$) between 5 and 50 Hz is 1.36% and 2.7%, respectively (see Fig. S4a below). The reason for which we only considered distances of 0.5 and 1m is because at higher distances the current density is reduced to a negligible value, as categorically shown by the Joule heat density vs. length profile shown in Fig. 5 of the manuscript, also reproduced as Fig. S4b below. It becomes evident that even at 50 Hz the skin depth variance affects current density at less than 3% as shown in Fig. S4a. Moreover, as the effective path of the current density flow is not affected neither the associated resistance R will be affected ($R=\rho\frac{l}{A}$). Therefore, Joule heating loses, ${Q\sim I}^{2}R$ will not be affected by AC frequency.

|  | 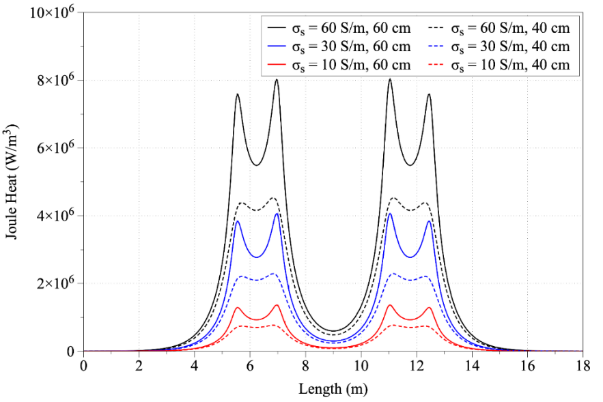 |
| --- | --- |
| **(a)** | **(b)** |

**Figure S4**. Schematic representation of (a) the current density relative difference and (b) Joule heat distribution as a function of electrode immersion depth and slag’s electrical conductivity.

A second firm indicator that Joule heating is practically independent of AC frequency, is related to the comparison between the slag’s ohmic resistance, R, vs. reactance, X. The latter receives contributions from slag inductive, X_L_, and capacitive, X_C_, reactance. The inductive reactance is defined as$X_{L}=i\omega L$, where ω is the AC radial frequency and L is the inductance. Slags are invariably semiconductive and their inductive impedance is virtually zero. (e.g., see in-vacuum values above 600 K in Herberlin, 2020[^5^](#_ENREF_5). The capacitive reactance is defined as $X_{c}=\frac{1}{j\omega C}$^[3](#_ENREF_3)^. In order to understand its contribution, one may consider a double layer of ions created around the electrode and, more specifically, on the interface between the electrode and the slag. Assuming a homogeneous electric field, we can approximate this interface as a cylindrical capacitor with capacitance, C, equal to

$C=2\pi\frac{\varepsilon_{0}\varepsilon_{r}}{ln\left( b/a \right)}L$

where α (equal to the electrode radius. i.e., 1 m) and b are the inner and outer capacitor radii and L is the capacitor length, in this case equal to the electrode immersion depth. As b decreases, X_C_ increases. Therefore, setting b to a very small value (with re. to electrode radius) of even down to 1 mm, at 5 and 50 Hz, X_C_ is equal to 1.75 and 0.175 Ω, respectively. To appreciate if these X_C_ values are of practical significance to Joule heating, they need to be compared against the slag’s ohmic resistance, R. To calculate R, the region between the electrodes (through which AC diffuses) may be approximated as a cylinder of radius r, equal to the distance between two electrodes (i.e., 6 m), of length at least equal to the electrode immersion depth. The ohmic resistance of this region would be equal to

$$R=\rho l/A$$

with $l$ the length of the cylinder and A the cross-sectional area, equal to 4πr^2^. Substitution of parameter values yields R =141.37$\Omega$. Comparing R (141.37 Ω) X_C_ (1.75 and 0.175 Ω at 5 and 50 Hz, respectively) reveals that R is always at least two orders higher than X_C_. Hence, Joule heating is most categorically AC frequency independent.

**References**

1 GmbH, V. S. *Slag Atlas*. Vol. 11 (1995).

2 Sibaki, E. K. *et al.* A numerical study on the influence of the frequency of the appliced AC current on the electroslag remelting process. *Proceedings of the 2013 International Symposium on Liquid Metal Processing & Casting* (2013).

3 Hayt, W. H. *Engineering Electromagnetics (Mcgraw-Hill Series in Electrical Engineering. Electromagnetics)*. (1989).

4 Davidson, P. A. *An introduction to magnetohydrodynamics*. Vol. 25 (Cambridge university press, 2001).

5 Herbelin, M. *et al.* Steel Slag Characterisation-Benefit of Coupling Chemical, Mineralogical and Magnetic Techniques. *Minerals-Basel* **10**, doi:10.3390/min10080705 (2020).
